# Transactions of the American Association of Obstetricians and Gynæcologists

**Published:** 1888-11

**Authors:** 


### SOCIETY REPORTS.

### TRANSACTIONS OF THE AMERICAN ASSOCIATION OF OBSTETRICIANS AND GYNÆCOLOGISTS.

The first annual meeting was held at Washington, September 18, 19, 20, 1888.

First Day—Morning Session.

The President, Dr. W. H. Taylor, of Cincinnati, called the meeting to order and delivered his annual address, in which he said: On the assembling of this association for the first time, it may be proper to demand a reason for its existence. The daily round of professional experience impresses the practitioner of medicine with the incompleteness of his knowledge, and to the man who sees in his avocation something more than a mere source of pecuniary profit there must arise the desire to know the yet unknown. No argument is needed to prove the assertion that the united effort of many is more fruitful than the inharmonious working of individuals; hence the propriety of co-operative organization. It may be admitted that the motive of our association is, first, our own advancement, yet it cannot be considered undue self-adulation if we believe that from such combined effort good must come to the profession at large, and necessarily through the profession to those who, above all others, are interested in the perfection of our knowledge and skill—our clients.

The activity of the past decade has given us so many important facts that it would scarcely be hyperbole to say that a new practice of obstetrics and gynæcology has been created in that time. But the very fact that there has been such progress, and that so much that but a few years ago was impracticable or even unknown is now feasible and well known, only stimulates us to better work and further advance; and, with the vast range of study and diversity of subjects now comprehended by the science of medicine, it is clear, beyond controversy, that advance can be made only by men directing their efforts to a limited field of work. Such, gentlemen, I believe, are sufficient reasons for our presence here to-day as the American Association of Obstetricians and Gynæcologists.

An opinion has large credence among the laity, and is indorsed by some members of our profession, that intentional abortion is a common crime, while statisticians and social scientists see in the decreasing fecundity of American women clear evidence of the truth of this charge. That some women are guilty admits of no doubt, but that it is so universal an evil as sensational literature, especially of non-professional origin, would indicate, I do not believe. Permit me to direct your attention to a more demonstrable cause for diminishing fertility. I need but remind you of the deleterious influence of social habits and circumstances over the functions and physique of women, and, consequently, how many fashionably-educated girls are incapable of completing the process of maternity. Those who are fond of charging American women with the crime referred to, cite to us the rich, the educated, the refined, as guilty, while their sisters who are less favored financially, and are humbler socially, bear their due proportion of children. Now, the first class are the physically feeble, the enervated, and therefore incapable of carrying the process of reproduction to its ultimate perfection. A lack of fecundity on the part of a woman is often due to endometritis, and is overcome by the cure of the diseased condition. Many cases of sterility after the birth of one child are due to inflammatory processes of the genitalia. Accept these statements as true, and a rational explanation of the asserted evil is found, which at once transfers it from the domain of ethics and morals to within the pale of our professional care, and at the same time vindicates those whom we look upon as the purest and best from the indiscriminate charge of the murder of their unborn children.

I believe the influence of the genitalia over the general system, especially in its nervous manifestations, has been exaggerated; that entirely too much attention has been paid to the genital organs in connection with the neuroses.

A subject of practical importance, urgently demanding attention from the obstetrical section of our association, is the constantly-increasing inability of women, who should be "nursing mothers," to furnish milk for their infants. Many women who have borne children are incapable of nourishing them; probably about 40 per cent. with us, and, according to Escherich and others, in Bavaria, 59 per cent. of mothers become incapable from physical causes of nursing their children within two months after delivery. In a large degree this is the result of habits tending to impair the integrity of the mammary glands.

Another topic well worthy of our most careful thought is, What can be done to mitigate the pains of labor? My impression is that, with the great majority of obstetricians, but little is done to lessen pain during a large part of the process of delivery. With the abundant therapeutic resources of the present day, we ought surely to divest this ordeal of the intensity of its pain. I trust by another meeting to report upon some experiments I am now making.

It has been said, as civilization progresses the tendencies of all human diseases has been to assume the neurotic type, and also that diseases become more complex in their manifestation. On the other hand, the recent advances in antisepsis allow the hope that some day we may eradicate the septic diseases of child-birth, and that our grand advances in surgery shall qualify us to cope successfully with what, a few years ago, would have been considered a helpless and hopeless condition. Science knows no sentiment, and we can cherish the achievements of the past only so far as they aid us for the future. Science almost every day brings a new surprise, so we may confidently anticipate advances and successes which shall as far surpass our present attainment as these do those of the past generation, and happy may we count ourselves that this association shall contribute to these beneficent attainments.

Dr. A. CORDES, of Geneva, Switzerland, sent two memoirs, which were read by the secretary. The first paper was on the "Treatment of Puerperal Eclampsia," and contained a report of four cases treated by the injection of bromide of potassium and chloral hydrate, with abstraction of blood over the mastoid processes by leeches. He advocated this treatment in preference to general bleeding, chloroform, and arterial sedatives. He also deprecated any interference with the genitalia, such as digital examination, passing the catheter, etc., which he thinks are liable to provoke convulsions.

The second paper was "The Treatment of Endometritis" by injections of pure nitric acid, and was illustrated by three cases in which this method was employed. The writer preferred this method of treatment to that by the curette, in cases where there were fungosities or other irregularities of the intra-uterine surfaces.

Dr. A. LAPTHORN SMITH, of Montreal: I would like to be permitted to say a few words on this very important subject. I think we should lay down three principles to guide us in these cases. Puerperal convulsions are due to mechanical pressure on the renal veins. If that remains too long, it will bring on uræmia. Uræmia continued long enough produces permanent damage to the brain. We should empty the uterus and take the pressure off the renal veins at the earliest moment we are sure such a condition is in existence. I have acted myself for two or three years on that plan, and have had no cause to regret it. The only regret I have is that during five or six years I adopted the method of waiting. Although none of the patients died, all of the children did, and one of the patients was in an asylum several years. I think if premature delivery is brought on under antiseptic precautions, we will in all cases save the woman, preserve her brain intact, and occasionally save the child. But the child is, however, a secondary consideration.

Dr. BYRON STANTON, of Cincinnati: I would like to ask if bringing on labor is not adding fuel to the flame; if it is not better to defer as long as possible the induction of labor, and treat the renal condition; place the patient in better condition for delivery. We know that convulsions do not always cease at delivery.

Dr. A. LAPTHORN SMITH: I do not think you should wait until the woman has convulsions. If you know that the urine is loaded with albumen, bring on labor without delay. Every moment of delay you increase the danger.

Dr. JOSEPH PRICE, of Philadelphia: The gentleman that has just taken his seat expresses a great deal of wisdom in his remarks. Mr. Tait calls attention to the frequency of such troubles in elderly primiparæ. It is claimed that it is exceedingly common in that class of patients.

"The Indications for Drainage in Abdominal Surgery" was the subject, of a paper by Dr. JOSEPH PRICE, of Philadelphia. He said the object of his paper was to point out the indications for drainage in abdominal surgery. At the present time, there is the greatest variance in the opinions and practice of surgeons upon this point, and some fixed rules, such as are laid down in other departments of operative surgery, are needed.

First: Is there danger from the retention of fluids in the peritoneal cavity? Second: Is the drainage-tube a safe means of obviating this danger? Third: If the tube involves danger, what are the comparative risks from its employment or omission? That the peritoneum will relieve itself of exuded fluids is well known; and it is also an established fact that the free use of saline cathartics will often arrest acute peritonitis. Experience has also shown that these resources often fail, and it is necessary to re-open the abdomen, irrigate the peritoneum, and insert a tube; this, too, in cases where no pus is found. It is a fallacy to believe that extensive adhesions are alone an essential indication for drainage, since free exudation can arise from severing slight adhesions and extensive bleeding follow slight oozing after the patient reacts. This latter fact was illustrated in a recent case in which bloody exudation, which seemed almost nothing at the close of the operation, flowed freely through the tube for ten days afterward. A great difficulty is presented in deciding a case to be simple, particularly in cases of inflammation of the tubes and ovaries. Indeed, no one can decide during an operation whether the inflammation is simple, purulent, or specific. Herein is an additional indication for the cautery used by Keith. Drainage removes the pabulum of infection. In case of very extensive adhesions and positive hæmorrhage, the value of the tube cannot be questioned.

The danger involved in inserting a simple glass tube in the abdomen is very small. The involvement of the peritoneum in the perforations of the Bantock tube has been urged as an objection to its frequent use. This danger may be entirely obviated by carefully packing the tube lightly with absorbent cotton, which also, by capiliarity, facilitates drainage. The tube should be rotated occasionally and slightly elevated. When kept clean, by frequently changing the cotton, with careful attention to that part of the incision through which it is introduced, there is no appreciable danger of infection. I have reached this conclusion after using it in extreme cases of pelvic adhesions, extra-uterine pregnancy, hysterectomy, pus tubes, and so-called simple operations. I have never had a single fatal result which could be attributed to the use of the drainage-tube, and in numerous cases I have seen serious trouble relieved by its introduction.

Irrigation is an important adjunct to drainage in abdominal work. It is of the utmost value in removing débris, clots, and shreds. Pouring water through the incision is not so efficacious as its introduction by a syringe, which makes an uninterrupted current. I place the nozzle of the syringe through the lower angle of the incision, and with two fingers retract the intestines, thus giving free exit to the current. In this way the entire abdominal cavity can be thoroughly washed out. I can not see any advantage in Professor Martin's method of drainage through the vagina. To close the ventral incision to make one in the vagina seems to me unnecessary and illogical. If drainage be decided upon, the site of the incision offers every requisite.

All chemical solutions are unnecessary and positively harmful. Such agents introduced into the peritoneum have done much mischief. The intestinal pain so often following operations in which solutions were applied is, in many cases, due to the adhesions formed in consequence of their use.

In deciding the important question, When shall the tube be removed? we should look to the character and quantity of the discharge. When this is clear and sweet and scant, the tube should be removed. It has been urged that the tube delays union and thereby increases the danger of ventral hernia. This I do not believe. A very long incision, getting up too soon, and discarding the bandage too early, are the most potent causes of this accident. Indeed, I believe the objections urged against the use of the drainage-tube are theoretical and deserve no consideration, in view of the important and serious perils eliminated by its use, viz., protection of the peritoneum from infection. The faith I have placed in it has been fully justified by the results attained.

Dr. Clinton Cushing, of San Francisco, presented a contribution to the study of "Pelvic Abscess." He defined a pelvic abscess to be any collection of pus in the cavity of the pelvis which exists in the tissues outside of the cavity of the hollow organs, such as the bladder, uterus, Fallopian tubes, and the rectum. He was becoming more and more convinced each year of the truth of the statement made by Noeggerath, that uncured gonorrhœa in the male is a very frequent cause of salpingitis. A recent case came under his observation, in which this theory was verified in every particular. A woman twenty-seven years old, had been seduced nine years before; since that time she had apparently led a moral life, but had suffered from chronic pelvic abscess, resulting in repeated attacks of pelvic inflammation. She had lately married, immediately following which all her symptoms became very much aggravated, while the husband himself contracted a severe urethritis, which resulted in perineal abscess. Her case becoming serious, he saw her and diagnosticated a probable pelvic abscess. Upon opening the abdomen the right Fallopian tube was found distended with pus at its larger extremity, and a pelvic abscess containing several ounces of pus. He removed both tubes and ovaries. A microscopic examination showed gonococci present in both tubes, mingled with the pus. The secretion of the urethra of the husband also contained gonococci. This case would seem to demonstrate that the woman had been infected with the gonorrhœa nine years before; that its specific character remained in the Fallopian tubes, causing frequent attacks of pelvic inflammation; that the husband had contracted the disease of the woman; that the sexual relations had aggravated the already existing disease of the woman, resulting in the formation of the pelvic abscess, which necessitated operation. She recovered without a bad symptom.

In another case, he opened the abdomen and found that a portion of the abdominal cavity below the umbilicus was an immense sac of pus, containing at least a gallon. A counter-opening through Douglas' pouch was made, and a drainage-tube drawn through the abdominal opening into the vagina. On the second day the bladder sloughed, the urine escaping into the pus cavity. An artificial vesico-vaginal fistula was formed, that the urine might drain from below, and thus enable the pus cavity to heal. At the end of the year the cavity was healed, the abdominal wound was closed, the vesico-vaginal fistula was repaired, and the patient restored to health.

Dr. Joseph Eastman, of Indianapolis: I wish to remark with reference to the drainage-tube and dilating trocar, that Professor Martin, of Berlin, has been using the ordinary dressing forceps, forcing it through the vault of the vagina, seizing the drainage-tube, and drawing it up in that manner. I have in that way been using the Bozeman dressing forceps, simply sharpening their points.

I was much interested in the paper of Dr. Price. I believe, in a publication of mine, I made the statement that I would drain upon the slightest excuse. I still hold to that. As to the question of permanent drainage, Dr. Wylie, of New York, speaks of establishing permanent drainage. I do not know how that is to be done. In my experience, and that of others, I find that, in at least a week, nature has covered in the tube with plastic material. In the first ovariotomy by Dr. McDowell, he allowed the ligatures to hang out of the abdominal wound. I am not sure but that it is about as good drainege as we ever had or will have. It is good capillary drainage. In my experience in fifty-one cases, I have not had a hernia except in one case. That was where I had to leave the drainage-tube in over a week. It seems to me the key to the situation is in bringing the abdominal layers into exact apposition. I am satisfied that we must not decide for either vaginal drainage or abdominal drainage exclusively.

Dr. E. E. MONTGOMERY, of Philadelphia: The members of the association are greatly indebted to Drs. Price and Cushing for their practical papers on a subject of the greatest importance to those practicing abdominal surgery. I take it, in the practice of this department, we are desirous of using no measures unnecessarily, and on the other hand of leaving undone nothing that is important for the relief and salvation of our patients. With this in view, it seems to me that it is exceedingly important that practical indications should be laid down for the treatment of such cases, to make sure the class of cases in which drainage is indicated and necessary, and outline those in which it is not required. In many cases the necessity of drainage may be obviated by the subsequent care of the patient. If we place the patient on a restricted diet, or diminish the amount of liquid, so that the pressure in the vessels will be decreased and they, instead of throwing out liquid will take it up, there will be less danger of peritoneal effusion. In the applications of the dressings, where we have reason to expect peritoneal effusion, if we apply pressure we will find the danger of effusion is lessened. Another important thing is the care of the drainage-tube, so that the secretions are not allowed to decompose in the peritoneal cavity. By the application around the drainage-tube of the rubber dam, a piece of rubber such as is used by the dentists, making a complete covering over the dressings, we can wash out the cavity without soiling them at all. The drainage-tube is objectionable where it is unnecessary, for the reason that it lessens the strength of the union in the wound. It is also for the reason that it increases the difficulty of keeping the cavity pure and sweet. There are cases, I think, in which vaginal drainage is preferable to the drainage from above.

Dr. H. O. MARCY, of Boston: There are two or three points in which I shall draw exceptions in the matter of detail to the gentleman who read the first paper. We certainly all understand that we want to get rid of deleterious, effete, poisonous material. The first question the operator places before himself is, that he does it by operative measures. If he is sure he does it, it is a gratuity to add the danger of the introduction of a tube which itself may carry in infection. If it is removed, I am sure he would agree with me that we have nothing to drain, because we have a healthy non-infective cavity. The question comes, Can we do this? If we are in doubt, Can drainage accomplish that which you desire? Unfortunately, we all have to answer that many times it signally fails when we have trusted to it. If we had an infectious tube, we would destroy the point of infection before we closed the wound. There is nothing better than to be certain that you follow back the infection to its very centre. If you have a doubt about the aseptic properties of the cavity, wash it freely with aseptic water. If you have not removed this the next question you ask is, Does the drainage-tube make your patient safer? The drainage-tube often fails. When the drainage-tube fails, then the only safety to your patient is reopening the cavity. I believe that, if we have to use drainage, we should drain in the direction of gravity and not against it; that is what drainage means if we understand it. I am strongly inclined to think favorably of vaginal drainage. I have designed a drainage-tube which I will be glad to present—two tubes lying in conjunction, with a diaphragm; with it you wash in either way, which I believe is of advantage.

Dr. ALBERT VANDER VEER, of Albany: I scarcely think there will be presented to us any subject of more importance than this of drainage in the pelvic cavity. It is with pleasure I refer to one man in this country, one working in the direction of drainage in pelvic abscess. Had he lived he would undoubtedly have carried the subject much further along and nearer to perfection than has been done since his death. I refer to the late Dr. E. R. Peaslee. At one time his percentage of recoveries was greater than any other man's. He excelled Sir Spencer Wells in his percentage of recoveries. His theory was to drain in the direction that it is natural for the fluid to flow. In introducing the drainage-tube at the lower end of the abdominal wound, I believe it is well to put in the suture in such a way that it can be tightened as soon as the tube is removed. I can only call to mind two cases in seventy operations where I had hernia occurring. One was because the patient got up too soon. In closing up the wound, bring peritoneum in connection with peritoneum, fascia with fascia, skin with skin. They must be brought together carefully. We must select, according to the cases, the form of drainage.

Dr. PRICE, in concluding, said: I meant simply to discuss actual disease. I was not dealing with given cases. I have studied most carefully the works of Dr. Martin, Tait and others. When I consider the mortality of twelve in seventy-two for pelvic surgery, I must say that I do think Dr. Martin needs a little further experience in pelvic drainage. If you refer to Bantock and compare his mortality record, I think you will consider that it is wise to practice the drainage of Bantock, of Tait, and of Keith, and not that of Martin.

In regard to hernia, you can pick them out from a class of operators that make long incisions. An incision that is long enough to admit two fingers is quite sufficient. You can do anything in the pelvis with two fingers that you can do with the whole hand. The capillary drain I do not look upon as of very great importance. The frequency of cleaning the tube is of paramount importance. You cannot get along without a nurse with special training. A word with regard to the aspirator. I used it a few years ago; I have no need for it now. It is a very easy matter to keep a tube clean, sweet, and dry. I would call attention to eighty-four cases done in the alleys and courts, without carbolic acid or bichloride of mercury, with one death in eighty-four. The nurses were all trained to clean those tubes. I have had no experience with vaginal drainage; the other has served me well. The tube is a little sentinel to show when hæmorrhage occurs.

AFTERNOON SESSION.

Dr. WILLIAM H. MEYERS, of Fort Wayne, presented a paper on the "Treatment of Suppurative Peritonitis," in which he asserted that he knew of no reason why the same general principles should not apply to the largest serous sac in the body, where life is in danger from the irritant nature of its inflammatory contents, as holds good when pus has formed in the brain, in the pleural cavities, joints, or cellular tissue; namely, that all these conditions are amenable to surgical interference at the earliest possible moment after we have arrived at an exact diagnosis. He distinctly emphasized that he did not believe that peritonitis was ever idiopathic or spontaneous in its origin; nor that the peritoneum was ever the seat of independent inflammation, but that it was peculiarly liable to inflammation of a secondary character, the irritant proceeding from without, as in wounds, or from one of the organs lying underneath the membrane; or from injuries to the abdominal parieties, rupture of hydatid, ovarian, or other cysts, the bursting of an abscess, perforation from the diseases of the hollow viscera; these being the most common causes. Regarding ovariotomy as the parent of peritoneal surgery, he presented a résumé of a few cases in support of the idea advanced by Mr. Barker, where ovariotomy was performed during the progress of acute peritonitis, and quoted Mr. Wiltshire as saying that he was able to show, many years ago, that the presence of peritonitis is not always a bar to operations. On the contrary, it may be the chief indication of the necessity for operation, being itself due to acute cystic changes, and subsiding only on their removal. Accepting this principle, he believed that attempts at radical cure might justifiably be made, in proper cases, even in the presence of peritonitis. Referring to treatment by the trocar and aspirator, he quoted from a letter from Professor Thomas Anandale, which is believed to be an account of the first authenticated case where the trocar and washing out of the abdominal cavity was resorted to as a curative measure in suppurative peritonitis.

Dr. E. E. Montgomery, of Philadelphia, read a paper on "Larapatomy in Peritonitis," in which he said: The dread of disturbing the peritoneal cavity has been so great as to well justify the assertion of Wagner that he and his contemporaries had been nutured in the fear of the Lord and the peritoneum. Peritonitis varies in its phenomena according to the condition producing it. It is recognized that the disease, in the idiopathic form, can arise from exposure to cold and dampness, in which the exudation is limited and likely to be serous. In other forms, exudation is more likely to become purulent, or there is an early secretion of pus. The pus may be free, or become encysted.

The largest collections are more likely to occur in the acute disease, and especially in the puerperal and metastatic forms. According to Bamberger, the exudation, in its beginning, is fibrinous, but it is evident that there is a primary pus formation, as the cases quoted by Abercrombie and Kaiser show where pus was found after thirty-six to forty-eight hours of inflammation. The removal of pus is as imperatively indicated as when it occurs in pleural or articular cavities.

The presence of pus is indicated by fluctuation, dull percussion, attacks of chills, hectic, cessation of spontaneous pain, sensitiveness upon contact and movement. The diagnosis may be confirmed by puncture with a hypodermic or small aspirator needle. Pain is not always present in peritonitis, though pus may be present in large quantities. Non-purulent effusion may be removed by puncture, but suppuration positively indicates laparotomy, as it allows of thorough evacuation of the pus, complete cleansing of the abdominal cavity, separation of adhesions, the removal of exudation, and perfect drainage. It also permits of thorough examination, and may enable us to discover and remove the cause of the inflammation.

In perforative peritonitis, the operation should be done in all cases in which collapse has not already taken place, though the result will be more favorable in the cases of traumatic origin. In these, the patient is usually healthy, tissues of the viscus are in good condition, and sutures are readily borne, should resection be required.

The class of cases demanding early attention are those occurring about the region of the cæcum. These cases arise generally from perforation of the appendix. It has been stated that 34 per cent. die within three days. In severe cases, laparatomy should be performed at once; and when gangrenous or perforated, the appendix should be amputated and the stump sutured. Lateral incision is preferable to median.

In no form of peritonitis has laparatomy proven more valuable than in the tubercular. Its value was discovered by accident, the operation being done under mistaken diagnosis. The favorable result is attributed to the removal of the serum which serves as culture-fluid for the multiplication of the bacilli.

Incision, in these cases, is preferable to puncture, as ascites does not recur after the former, while it rapidly accumulates after the latter.

In conclusion, peritonitis should be regarded as a positive indication rather than a contra-indication for operation.

Death, in the cases of which we have spoken, is certain; we have seen that the operation may save the patient. Why hesitate to give the latter some chance of recovery?

Dr. Henry O. Marcy: I consider it a privilege to rise and express my thanks to Dr. Montgomery. It is now nearly two years since I opened the abdomen in one case for exploration, because of a considerable thickened mass post uterine. The patient had been six months under observation and gradually became worse. The diagnosis had not by any means been certain. I do not suppose it had occurred to any of us that she had tuberculosis, but the exploration showed that the whole peritoneum was studded with those little masses which are so familiar to you all. There was in this instance no especial amount of serum. The abdominal cavity was somewhat carefully washed out with a sublimate solution. Before closing the cavity, I cut off a little portion and submitted it to Dr. Nelson, who made not only a careful microscopic study, but cultivated the bacilli by artificial process, so that there was no doubt about the condition. But, strange to me and to you, the patient from that moment began to improve and is now comparatively well.

Dr. Clarke: Dr. Marcy invited me to see a case in which exploratory incision was done very late. Had it been done earlier I think it would have been successful. I have had an opportunity of seeing a good many such cases since, and I hope, and have encouragement to believe, that it is a feasible operation. Not long since, I saw a young girl who had evidence of phthisis and bacilli of the intestine, who, after a very small exploratory incision and draining off all the serum, seemed to get well in a short time. There was considerable serum in the case.

Dr. Ricketts, of Cincinnati: The discussion of the treatment of peritonitis by abdominal section has not taken the stand here that I hoped for. It is a very important subject, and I feel that the death-rate of peritonitis is going to be greatly lessened in the next few years by abdominal section. The trouble in these cases is to have the general practitioner understand the gravity of these cases early enough. It is not always the abdominal surgeon who sees the case in the beginning. Many times a bad result is counted against the surgeon, not for any failure on his part, but by failure to recognize the case at the proper time. I feel that it is the duty of every abdominal surgeon to do what he can to get the general practitioner to understand when to call assistance in these cases. I think it is our duty to give them to understand the importance of early interference in these cases.

Dr. Thomas Opie, of Baltimore: A remark has brought to my remembrance a case—a single one in my experience—in which I was called in by two physicians; I think on the fourth day after labor. The patient was then in a very bad condition, in a very critical state. I asked immediately if there was not some traumatic origin of the difficulty. They insisted that, so far as they were able to judge, there was none. I insisted on a digital examination, and found there was a rent in the right side of the cervix, in which, by some very remarkable accident—possibly one of the blades of the forceps was not introduced into the os—the uterus was torn very severely. Four fingers of my hand would go into a deep rent. I said to the gentlemen that I thought she was almost moribund, but that it was worth while to try something; it was a certainty that death would ensue, but something was left and that was laparatomy. Appreciating the gravity of the situation, it was insisted by the friends that we should give this one chance. I opened the abdominal cavity and found a large quantity of serum and many adhesions. The patient died ten or twelve hours after the operation. I mention the case because it was one in which I think it was right to give her the one chance out of a hundred.

"The Relation of the Abdominal Surgeon to the Obstetrician and Gynæcologist," was the subject of a paper by Dr. A. VANDER VEER, of Albany. He said:

Of those who remember the crusade which was early made against ovariotomy, which was but the beginning of abdominal surgery, and who cherish the belief that the latter is not yet a distinct part of general surgery, the question is asked, "Why is it that special works are being published on abdominal surgery alone, and meeting with ready acceptance by the profession?" Abdominal surgery may be said now to be a part of the specialties of obstetrics and gynæcology. Has not the work of such men as the late Professors S. D. Gross, Tait, Parkes, Sands, Weir, Senn, Greig, Smith, Bull, and others now living, demonstrated the fact that abdominal surgery has become a specialty? If we inquire into the manner of its development, we find that it has been the outgrowth of work done by the general practitioner. The elder Gross once stated that his experience and observation had taught him that they made the best specialists who, having engaged for a number of years in general practice, finally confined themselves to that special department for which their studies, inclinations, and clinical observations and experiences had best fitted them. These words apply with peculiar reference and force to him who attends to practice as an abdominal surgeon. The operation for removal of a simple uncomplicated ovarian cyst is probably the easiest operation that occurs in all the range of the major operations in surgery; but when we come to the operation, embarrassed as it may be by all the complications now known to us, it becomes one of the most difficult of all operations. Notwithstanding all that has been done within a short time by obstetricians, gynæcologists, or abdominal surgeons, to advance abdominal surgery, we are yet at the halfway house on the road leading to complete success, for not yet is made clear the best manner of operating, the best place for the operation, or the best way of dealing with all the various complications. Our entire profession must yet be taught much that pertains to abdominal surgery, and it must to a great extent be done by men of creative genius. What we need is not the elaborate presentation of theories, but the offering of practical papers that will give facts, and the accumulated wisdom and experience of honest workers.

Specialism in society work and in journalism should be encouraged by the profession at large. As abdominal surgeons we should move carefully, but at the present time we must be active and on the alert to make use of the constantly increasing material that presents, and from which to draw our practical deductions. In this new society we have formed a combination that illustrates well that trite but true saying that in union there is strength. The work of the obstetrician is the most arduous, important, and responsible of any part of our profession. The obstetrician and abdominal surgeon should work in perfect harmony. The latter bears to the former much the same relation that the general surgeon does to the neurologist in his brain surgery. The obstetrician, like the neurologist, will make his diagnosis, but the surgeon will often be called upon to do the operation. The work of the abdominal surgeon will necessarily lead him into the field of gynæcological practice—that is, the major portion, not the minor part, of gynæcology. There are many good gynæcologists who are successful in everything that pertains to their specialty aside from doing an abdominal section, and this they assign to some one else. I believe it requires years of the hardest study and effort to become a thorough abdominal surgeon; the number must ever be somewhat limited.

Finally, is it not a fact that there is some danger that abdominal section may be carried too far if made too much of a specialty? Have we not in this society the conservative combination?

Dr. S. C. GORDON, of Portland: I hardly know how we can discuss a paper like this except to assent to what has been said. The paper has certainly covered the ground. A discussion, in order to be interesting, must take a departure from the subject discussed. Abdominal surgery in my opinion is now taking quite a different turn from what it formerly did. Formerly we believed that everything of this nature must be done in a special hospital, with special accommodations. My own experience justifies me in saying that, while we must take every possible precaution, we must not allow a patient to die because we can not remove her to a special hospital. The question of special hospitals is no longer a sine qua non.

Professor W. H. WATHEN, of Louisville, read a paper on "The Surgical Treatment for Lacerations of the Perineum and the Pelvic Floor." He spoke especially of the surgical treatment of lacerations or injuries of the muscular and aponeurotic structures that form the floor of the pelvis. He said there is probably no other subject in gynæcology about which so much has been written that is of no real value, and that a relatively simple operation had been made to appear so complicated that it is seldom correctly performed.

He said that the muscles and the fascia in the perineum give it strength, and, when they are lacerated, no operation that does not primarily tend to reunite the torn ends is logical, or will be followed by permanently good results. We may have prolapsus of the uterus, with rectocele and cystocele, resulting from subcutaneous rupture of these structures, with no laceration or injury of the mucous membrane or other parts of the perineum. This condition is usually not diagnosticated by the attending physicians, and the woman is subjected to various plans of treatment to hold the parts in position and relieve the annoyance from pressure, weight, etc., all of which give but little relief ; nor can she recover, except by an operation to bring together and reunite the torn ends of the muscles and fascia.

Dr. HENRY O. MARCY, of Boston, read a paper upon "The Perineum, its Anatomy, Physiology, and Methods of Restoration after Injury." He reviewed carefully the teachings of the text-books and the views held by various authors whose writings are found scattered through the medical journals. These he supplemented by a series of photographs of dissections of his own work, from which he verified, in large measure, the teachings of Savage. He especially emphasized the importance of a better general knowledge of the anatomy of the pelvic structures, and pointed out where the teachings of many of the text-books are in error in important particulars

SECOND DAY—MORNING SESSION.

Dr. W. W. POTTER, of Buffalo, reported a case of "Double Ovariotomy during Pregnancy; Subsequent Delivery at Term."

Dr. VANDER VEER said : Dr. Potter has presented just that kind and class of cases we ought to have given us. This is an exceedingly rare case. I have looked up the subject somewhat, and find ninety-six cases reported where the operation has been done during pregnancy—not *double* ovariotomy. It is an operation perfectly justifiable. The condition of pregnancy does not seem to interfere very much with abdominal operations. The case he referred to I operated on about three months ago. I had a letter from the physician in charge saying that she is progressing well, but I do not care to have her case go on record yet.

Dr. E. E. MONTGOMERY : I was very much interested in this case of Dr. Potter's. Two years ago I read a paper on Ovariotomy during Pregnancy. I was able to find a record of sixty cases, and showed that pregnancy had very little influence upon the morality of the operation. In all those, there was not one in which the operation was done upon both ovaries. In the report of Dr. Mundé, I think, it was claimed that a woman was not likely to go through gestation with both ovaries removed. This paper of Dr. Potter shows that this belief is erroneous. I had thought, until Dr. Potter made this report, that my case was the first.

Dr. MAXWELL, of Keokuk: There is one other point that is interesting—that is, that the patient operated upon nursed her child after gestation had been completed; another fact, that lactation is not a substitute for menstruation.

Dr. BYRON STANTON, of Cincinnati, read a paper on the subject of "Induced Premature Labor." He referred to the arguments which have been urged against this procedure, based upon the ground of its mortality and usefulness—arguments that would apply with equal force against all obstetric operations. He thought that, like the obstetric forceps and version, it would tend much to do away with craniotomy and Cæsarean section. He thought that the arguments that the mother is ex-exposed to some risks that she does not encounter at term, such as imperfect development of the uterus, incompleteness of the changes which nature effects in the maternal passages during pregnancy, the frequently delayed detachment of the placenta, more frequent malpresentations, etc., were more than met by the advantages gained by the smaller size of the child, and more yielding and compressible head, and consequently shorter and safer delivery.

He also referred to the arguments against induction of labor, on account of the great infant mortality, quoting some recent writers as holding that such intervention is so dangerous that the operation should give place to Cæsarean section—an operation that has rapidly reduced its mortality. He thought the conclusion not justified by statistics.

The points involved in determining the question as to the resort to the operation are:

1. Whether the case is one in which delivery at term will involve the death of the infant, and subject the mother to much danger and suffering, and yet permit delivery at seven or eight months;

2. Whether the conditions are such that the life of the mother will be imperiled by continuance of pregnancy to full period of gestation, by hæmorrhage, or some intercurrent disease, or by aggravation of some chronic ailment which can be averted or removed by earlier delivery; and,

3. Whether there is a history of infant fatality in several successive pregnancies at some regular time after the age of viability.

He considered the methods of inducing labor, of which there are many.

Medicines acting through the general system were condemned. All oxytocics are unsafe for inducing labor, so also are those methods that act by reflex irritation. Some method acting directly on the uterus is to be preferred, and only those are to be thought of that respect the integrity of the membranes. Electricity in its various forms has proved disappointing. The hopes that were at one time entertained have not been borne out by experience.

Kiwisch's, Kluge's, Cohen's, Hamilton's, Lehman's, Schiele's, Krause's, and other methods were described, preference being expressed for Krause's method—the introduction and retention of a flexible bougie between the membranes and uterine walls until uterine action is excited.

In placenta previa he would use a tampon in the vagina, or a Barnes dilator in the cervix, until sufficient dilatation is effected to permit version.

All methods that bring on uterine action by evacuation of the liquor amnii are to be avoided.

After labor, the child demands especial attention. It must be kept continuously warm by artificial heat. The bath usually given to the new-born *must be omitted.* Early feeding must be resorted to.

Dr. THOMAS OPIE: I think the attention of physicians ought to be called to the importance of the principle of taking advantage of that period of intra-uterine life in which the sutures are yet wide and the bones not well developed, and in which we can have this very desirable condition of molding as the head is passing through the canal. It is remarkable the ingenuity with which nature molds the descending head to conform to the channel through which it must pass, but this is a process which requires an amount of time proportionate to the difficulties to be overcome. If the contraction is great, the time must be lengthened. If the contraction is too great, then delivery must be impossible if the patient has gone on to full term. I wish to speak of great importance of taking advantage of the undeveloped condition of the head of the child. We will find the shape of the head indicating the direction of the force, and, so distinctly pointing out what has been the difficulty and cause of delay. We have to deal with two distinct classes of cases. In one class, after inducing labor, we can wait for Nature to carry it through, but, in cases of eclampsia and placenta previa, delivery must be accomplished at once. Still we should take advantage of the condition of the head, for, with the forceps upon it, we find it still qualified to adapt itself to the canal, but in a better way than at full term. In cases of eclampsia, I think many lives are lost on account of delay. It has not been my experience that the operation of dilating the uterus is either difficult or dangerous. The introduction of hard-rubber dilators will furnish the beginnings. It will soon be found that, after introducing one after the other, the contractions begin, whereas, with the bougie, it might be delayed two or three days. I merely introduce these as a forerunner of Molesworth's or Barnes' dilator, until I can get in two fingers; and then I am able to introduce Taylor's narrow-blade forceps, and with these dilate the os sufficiently to apply the Tarnier forceps.

I would emphasize what has been said with reference to the question of ergot. There is one point about ergot which should be thoroughly established in the minds of the whole profession—that there is great danger to the child from its tonic action, and, as far as the mother is concerned, we start a force that we can not control.

Dr. A. L. Smith, of Montreal: I wish to urge the induction of premature labor in bad cases of vomiting in pregnancy. I have a record of eighty cases of death in this comparatively simple condition. I know of one large family of beautiful girls in Montreal, every one of whom was born at seven months.

In a paper entitled, "Is the Frequent Use of Forceps Abusive?" Dr. Thomas Opie, of Baltimore, said: There is a remarkable unanimity of opinion in the teaching of recent text-books, medical journals, and medical societies on this subject.

They favor the more frequent use of forceps, and condemn the so-called expectant management of labor. The axiom "meddlesome midwifery" has beaten a retreat before the clamor for the forceps.

The name of forceps points to its most striking characteristic, its prehensile power. Three distinct eras may be traced in this branch of medicine, having their pivotal influence in the forceps.

1. The introduction of the original forceps in 1700; 2, the important invention by Levret of the pelvic curve in 1747; and 3, when in our own times, 1877, Tarnier invented the traction-rod attachments to the forceps.

Baudelocque arranged methodically the pelvic positions and presentations in 1791. Prior to that time the sovereign powers of the forceps were greatly abused. Their introduction was a matter of haphazard and convenience. Even in the days of Ramsbotham there was a bitter controversy as to which was preferable, forceps or craniotomy.

A recent writer says "the forceps is simple in construction, easy of application, wonderful in power."

The successive stages of their development have, on the contrary, made them far less simple in construction; they have a wider range of application and wonderful powers for life-destroying as well as life-saving.

All surgical operations are dangerous; it is equally true that elements of danger lurk about all cases of artificial labor.

To substitute traction for contraction; to introduce and use instruments in the genital tract; indeed, to substitute art for nature's peculiar and inimitable methods, is always dangerous.

The most unanswerable argument against frequent use of forceps comes from the gynæcologists. Good surgeons are relatively few, and delicate and dangerous operations in that branch are relegated to specialists among them; but most practitioners think themselves able to cope with any forceps case. If skill in the use of forceps did not exist prior to Baudelocque's classification, how can we accord this virtue to operators with the forceps in these days, who are ignorant of the fundamental principles and admirable system upon which the present science of obstetrics rests?

The opportunities for the student of obstetrics are meagre; and granting he is thorough in the theory of this department, he forgets it before he is intrusted with a practice. No wonder if he makes a medium-rate obstetrician, and sneers at the mechanism of labor.

To determine between the high forceps operation and version, to diagnosticate or rectify position above or at the brim, to apply the forceps accurately and conduct safely the little passenger through the passageway, are among the great achievements of medical science.

The high forceps operation is a critical one, and the operator should be chosen with as much care as for an ovariotomy.

The low operation is not so simple as to be left to "any tyro."

For practical purposes, it answers to designate all kinds of forceps, in the order of their seniority of invention, as follows: the straight (Chamberlyn), curved (Levret), and traction-rod (Tarnier).

It is high time to turn a deaf ear to the many names of individuals attached to forceps because of various modifications of lock, blades, or handles. They obscure the horizon of the subject; they distract attention and confuse the object, which, like that of the practice of medicine in all its departments, is life-saving, curative.

There is but little need for straight forceps. The traction-rod forceps alone serves our purposes with satisfaction, at and above the superior strait.

The welding and unification of the traction-rod and curved forceps in Tarnier's latest style well-nigh disarms criticism. It is a great achievement; one on which we may congratulate this age.

Think of the struggle, and pity the sufferings of the man, woman, and child as the operator toils away with the straight forceps at the inlet. The pelvic curve added would only qualify, not remedy, the situation.

Still another modification, and science has triumphed. The traction-rods, with relative ease and far less force, pilot the head through the upper narrows, and thence into the safer region of the low operation.

If we could only persuade operators of the salvation of traction-rods covering all the indications for traction at the superior strait; of making traction, during the pains, rythmical, direct, slow; of supplementing, not superseding, nature; of utilizing all the vis-a-tergo and only just enough of his power to bring the combined forces to the normal, and that all above that is abuse.

If we could get them to be careful, to halt, prospect and protect the sphincters of the uterus and vagina, and to beware of rapidly unloading the organ, we would have a reform that would be as life-saving stations all along the passageway.

I am fully convinced of the ability of the forceps to help. I protest that they should not be used regardless of the factors of delay.

I plead for their judicious use.

As to the indication that the os should always be dilated or dilatable, judgment often goes to protest.

About two years ago, I was in attendance upon a most tedious, nervous, and impatient primipara, in whom the os was only three-quarters dilated ; after a boisterous struggle of two nights and two days, slowly, almost imperceptibly, the dilatation increased. The patient's-qualifications for abuse, however, grew more rapidly ; I came in for a large share of it. She demanded chloroform and instrumental aid ; the husband seconded her claim. The traction-rod forceps was applied ; she was delivered in two hours under chloroform. A febrile trouble and tardy convalescence ensued ; some months afterward she became the patient of a clever gynæcologist. His operation told the tale of the "blundering obstetrician." I have never had an opportunity since to call the patient to an account.

One of the most important requisites for a good obstetrician is "to know how to wait and do nothing." In the first stage he must wait a long time, especially in primiparæ, before using forceps; indeed, he is not warranted in resorting to it, except the mother or child is in jeopardy.

For the most frequent use of forceps is in the second stage, and we are told "any tyro" can perform this operation. Nature can generally do it better and safer, if not so quickly. Time and patience can not be too much extolled as virtues during the whole of labor.

Let the attendant occupy the position of "watchful expectancy." Remove reflex disturbance, sustain by proper food, guard against fatigue, secure rest, assure and cheer, utilize the simpler methods of assistance ; let nature do all she can before we resort to forcible measures. I have lately limited the number of my forceps application by manual pressure, according to the plan advised by Kristeller of Berlin.

This, and other manual helps, are more in accord with nature's laws, and should be made use of. Instead of using forceps to curtail the sufferings of labor, we had better consider the very great efficacy of morphia, chloroform, and chloral.

As an isolated indication, it is doubtful whether pain ever warrants forceps use, nor would such cases necessarily end with less suffering after immediate delivery.

It is a common occurrence for women to demand that the accoucheur shall do something to help the physiological phenomena of labor. Dr. Robert Barnes denounced this as an old and bad practice.

Anæsthesia is the best treatment for cases of excessive nervousness and emotionalism in labor, not the forceps, as has been advised. Chloroform during the pains often makes labor of this sort more normal.

The nervous element is often misleading; it is not always the patient who is most noisy who suffers most, nor is it safe to be influenced by her cries for more chloroform or the forceps.

The loss of time by an accoucheur is not to be accepted as a warrant for resorting to instrumental delivery.

The forceps is a life-saving instrument for both mother and child, whether we are dealing with the first or second stage of labor, provided it is used judiciously and skillfully.

There is much difficulty in obtaining exact statistics as to the frequency with which the forceps is used. The figures vary with the country and with the operator. Ploss' tables show a wide difference in frequency of application in German, Swiss, and Russian maternities. In an aggregate of 33,054 labors, the forceps was applied 1,975 times, or one in thirty applications.

Ploss' tables for England make the least number of applications for any operator, as one in 3,878. The operator with the greatest relative number was one in 7.8. Jacquemier's tables give 20,517 labors and ninety-six forceps cases, or one in 214.

These tables embrace a period from 1792 to 1862. Those were the days of the swaddling infancy of obstetric knowledge, when method was just inaugurated by Baudelocque. Still they are useful in the light of history, in setting forth the wide range of opinion as to the frequent use of the forceps in those days.

Galabin says no one would now recommend so sparingly a use of forceps as one in 200. He says that the statistics of St. Thomas' Lying-in Charity appear to show that a forceps rate of one in sixteen to one in eighteen deliveries does not endanger the mothers; but wider statistics are desired.

The Maternity Hospital, Philadelphia, shows the number of forceps applications in 1886 to have been about one in eight.

The Columbia Hospital, Washington, D. C., has had an average of about one in twenty. At the Maryland Maternity Hospital, Baltimore, we have used forceps 142 times in 1,495 deliveries, or one in 9.4.

The number of forceps applications at Vienna Lying-in Asylum during the past year was 2 per cent.

In my private practice, including consultation work, the average has been one in 10.5. I have found by inquiry of many practitioners that their average in private practice ranges from one in four deliveries to one in twenty-five.

It is to be deprecated that there is not a greater tendency to specialism in obstetrics. No man, ignorant of the mechanism of labor, and who does not know the rules for using the forceps, should ever undertake a high forceps operation. That clumsy, untutored, and unskilled hands do harm with the forceps even at the lower strait cannot be denied. As long as this unfitness and want of training for obstetric operations exists, it will be humane to withhold our advocacy of a still more frequent use of forceps.

Nature has been defined as "the good will of God expressed in facts." We should crave her facts and imitate her. We will be adverse to substituting art for nature, so long as man is inferior to his Maker, except we act because of danger to mother or child.

We will not detain you with the enumeration of the many accidents and injuries which befall the mother and child at the hands of the forceps. Suffice it to say, they embrace a large part of every recent work on gynæcology.

We sadly need statistics to settle the wide differences among obstetric operators with the forceps as to the frequency with which they may be used. Our judgment is that the average of one in fifteen or sixteen applications of forceps, as suggested by Dr. Galabin, is a fair one. The pendulum, having properly left the position of too great infrequency, has gone to an unwarranted extreme in point of frequency.

The obstetrician would do well to be found in the good company of the physician in the prevention of disease, the surgeon in his conservatism, and in line with the modern sanitarian.

Dr. Joseph Price, of Philadelphia: For years I condemned the use of forceps in unqualified language. During my hospital experience I singled out 300 cases of labor, in twenty-five of which forceps had been used in previous labors. In none of the twenty-five cases could I find the slightest indication for the use of forceps. I know that in skilled, trained hands it is a valuable instrument. I am glad the author has advocated the Tarnier forceps, and has called attention to the Taylor narrow-blade forceps. In one case—a very ugly patient—in five successive labors evisceration had been performed. She finally agreed to submit to induced labor, and thereafter three living children were delivered with the traction-rod and Sims' forceps. The Sims forceps have a great advantage in the greater diameter of the blade. I have gone to three series of 100 cases without taking my forceps out of the bag. In a discussion on the use and abuse of the forceps in our obstetrical society, one man admitted that he could not afford to attend poor women for five or ten dollars and not use forceps. It was five or six months before he came back to the society, so thoroughly was he rebuked.

Dr. FRANKLIN TOWNSEND, of Albany: It is important to bear in mind that in labor the first stage is governed by the sympathetic nervous system; in the second stage, by the cerebro-spinal system. I do not believe the second stage should be allowed to continue longer than four hours. The exhaustion due to the strain on the cerebro-spinal nervous system is such as to warrant a speedy termination of labor.

Dr. J. H. SCARFF, of Baltimore, reported a case of "Intraperitoneal Hematocele with Suppuration." A saleswoman, aged twenty-five years, was injured by the falling of a box from an upper shelf in a store, striking her upon the left side of the abdomen. Inflammation with subsequent exudation ensued, after the lapse of some months after the injury. During the progress of the case an aspirator needle was inserted, and a pistonful of disintegrated blood secured. About six months afterwards he performed laparatomy and removed a large basinful of suppurating clots. The patient made a speedy and good recovery.

He summarized the points in the case as follows:

1. Pelvic inflammation and exudation, simulating true pelvic tumor, resultant from an injury.

2. Rupture of a blood-vessel in the peritoneal cavity while on duty subsequently in a store, and formation of an intraperitoneal hematocele.

3. Rapid suppuration of blood clots due, probably, to infection by the aspirator needle.

4. The extent of the provisional sac for the retention of pus in the peritoneal cavity is noteworthy.

THIRD DAY—MORNING SESSION.

Dr. THOMAS J. MAXWELL, of Keokuk, presented a careful résumé on the subject of "The Diagnosis and Treatment of Uterine Fibroids," including the pathology and causation of the disease. He dwelt upon the difficulties of diagnosis, and called attention to the liability of its being complicated by co-existing retro-deviations of the womb. He gave his own experience narrating his cases, and pronounced in favor of ergot, electricity, and the knife, as affording the rational methods of management of uterine fibroids.

HAMPTON E. HILL, of Saco, reported "An Unusual Case of Subserous Uterine Fibroid—Operation." The tumor measured in length sixteen inches; in width, fifteen inches; in thickness, ten and one-half inches. The weight was forty-seven pounds.

Dr. EDWARD J. ILL, of Newark, read a paper on "Desmoid (Fibroid) Tumors of the Abdominal Walls." He cited two cases, in one of which one hundred square centimetres of peritoneum were removed. In both cases the peritoneal cavity was opened. One case was operated upon by the excellent method of Sänger. Both cases recovered. The writer then spoke at length of the anatomy, pathology, etiology, symptomatology, diagnosis, and treatment of these rare forms of tumors. He drew especial attention to the symptoms produced by the contraction of the abdominal muscles on the tumors. He dwelt at length upon the differential diagnosis between this form of tumor and enlarged spleen, enlarged kidney, subcutaneous or subperitoneal tumors, uterine fibroids, solid tumors of the ovary and round ligament, adherent displaced kidneys, cyst of the urachus, dermoids of the abdominal walls, etc.

The prognosis of true desmoid is good. He has collected twelve cases of this form of tumor occurring in the United States. Ten of these occurred in females between the ages of twenty-three and forty-one years. In five of these cases, the origin of the tumor was given as the fascia transversalis. In six cases, the tumor was so closely adherent to the peritoneum that it became necessary to open the peritoneal cavity in removing them. In two of these, excision of a large portion of the peritoneum became a necessity.

Dr. CHARLES A. L. REED, of Cincinnati, presented a paper on "Fibroid Tumors of the Abdominal Wall." He noted the dearth of American and English literature on tumors of this character. After accumulating his data, however, it became apparent that, to dispose of the entire topic, it would be necessary to discuss (*a*) fibromata, (*b*) lipomata, (*c*) myxo-lipomata, (*d*) cysto-carcinomata, (*e*) neuromata, and (*f*) hydatid cysts. A discussion of these subtopics, however cursory, would result in the production of an essay too voluminous for the occasion. As a consequence, and in consideration of the fact that his own observations had been limited to five cases of tumor of the abdominal wall, all of them fibroids, he determined it expedient to restrict his paper to a discussion of that variety. He considered the subject under the following heads: History, etiology, pathological anatomy, symptoms, development, influence of pregnancy on the nutrition of these growths, duration, prognosis, diagnosis, and treatment. He presented the subject of treatment under three heads—exploratory incision, total extirpation, and electrolosis—reporting his five cases in detail. The conclusions to which he had been forced by a study of fibroids of the abdominal wall, and by his experience in their treatment, were as follows: *First*, fibroids of the abdominal wall, however small, benign or indolent, are liable to take on active growth; *second*, when small, their anatomical relations are unimportant and their extirpation can be accomplished without serious risk to the patient, and should be advised; *third*, when permitted to grow large, their anatomical relations become very important, and their extirpation can be accomplished but with hazard to the patient; *fourth*, when in cases of large tumor, extirpation should be undertaken only where the exploratory incision demonstrated its feasibility; *fifth*, there should be no hesitancy in abandoning an operation when exploratory incision has shown it to be dangerous, because the primary incision may be of benefit to the tumor; *sixth*, in cases of manifestly deep-seated tumors with extensive peritoneal attachment, electro-puncture should be tried before extirpation is undertaken.

Professor GEORGE H. ROHÉ, of Baltimore, read a paper on "Diseases of the Skin, Associated with Sexual Disorders in the Female."

Dr. FRANKLIN TOWNSEND, Albany, presented the subject of

### EXTRA-UTERINE PREGNANCY: ITS PATHOLOGY.

Much of interest has been already published upon the subject of extra-uterine pregnancy, both of a theoretical, as well as of a practical, nature, but reference to results from broad studies in comparative anatomy, is but scanty. In tracing out pathological conditions, we should, of necessity, refer to histology and physiology, for without these scientific aids to our study, we lose sight of the finer shades of beginning morbid processes from absolutely normal ones. We should begin our study of the pathology of ectopic gestation by comprehending the physiological functions of the organs most intimately involved. The pathological processes following may then, I think, be more distinctly and definitely traced. Most authorities are agreed that the functions of the Fallopian tubes are, first, to transmit the ovum from the ovary to the uterus, and, second, to permit the passage of the spermatozoa from the uterus in the direction of the ovary. There are a few, and the best of authorities, who differ from this view.

Of the *Physiology of Impregnation of the*

*Ovum*, he said: The seat of contact between ovum and spermatozoon has not yet been determined with absolute certainty. Many of the best authorities differ in their conclusions upon this subject. Coste's observations seem to prove that fecundation is almost always effected, either upon the ovary, or in the part of the tube nearest the fimbriated extremity, inasmuch as he maintains that the ovule spoils very quickly when it enters the tube without previous fecundation. Regarding the functions of the tube and ovaries, Mr. Tait has proven, conclusively, that ovulation can and does take place before, during, and even after, menstruation ceases; also, that the changes in the ovary at puberty are simply vascular, and that those in the tubes at this period are vascular, muscular, and epithelial, and that the change of greatest importance is in the functional movement of those accessory organs. Ovulation, then, and menstruation are necessarily co-incident. If, then, ovulation continues intermenstrually when the tubes are quiescent, what becomes of the ovum when the ovisac ruptures? Mr. Tait says: "I believe that the ovum falls into and perishes in the peritoneal cavity in by far the greater number of cases, and that the passage of it into the uterus occurs only in a small minority of the ova produced."

Accepting, then, the views of the majority of the authorities, that fecundation usually takes place either in the tubes, or on the surface of the ovary, or even in the Graafian follicle, or possibly, as has been intimated by Parry, in the peritoneal cavity, and granting the admirable stand taken by Tait, it seems that, *first*, fecundation of the ovum takes place more frequently than is supposed. *Second*, that this being the fact, many sterile women, that is objectively sterile, who never complain of pain or ache, who ovulate and menstruate with greatest nicety and regularity, and whose general health is perfect, may frequently have fecundated ova, which, like non-fecundated ova, may drop into the peritoneal cavity and perish, because the soil there is unpropitious for their development. *Third*, that occasionally, but rarely, this same peritoneal soil, if permitted to use such a term, does present a favorable site for the development of the fecundated ovum, and what is called "primary abdominal pregnancy" results. *Fourth*, this propitious site may be due to the old peritoneal inflammatory troubles, which may be so slight, indeed, as to have never given rise to suspicion of their existence. Such nesting spots in the peritoneum for the development of the young fecundated ovum, though occurring not so frequently as those inflammatory changes in the tubes, causing desquamation of the ciliated epithelium, and thereby tubal pregnancy, as Mr. Tait so ably advocates, are, nevertheless, a factor of causation of the so-called "primary abdominal pregnancy."

*Ovarian Pregnancy.*—So long ago as the latter part of the seventeenth century, St. Maurice demonstrated a case of ovarian pregnancy. A number of cases of this very rare condition are now on record. Rupture of the sac takes place early, though when the sac walls are reinforced by inflammatory adhesions to the peritoneal coverings of adjacent viscera, gestation at full term may be reached.

*Abdominal Pregnancy.*—As has been shown, fecundated ova frequently drop into the abdominal cavity and perish. Occasionally it happens that their growth may continue for an indefinite period. The pathological changes occurring in this form of primary abdominal pregnancy must be distinguished from those that take place in what is termed "secondary." In the one instance we have so minute, soft, fragile, and delicate a corpuscle deposited in the peritoneal cavity that one could not well imagine any grave inflammatory results accruing from its immediate presence. This being the case, then, the contiguous abdominal organs will not be injured by its ulterior development. On the other hand, in the secondary form of intra-peritoneal pregnancy, we have a voluminous product of conception suddenly thrust upon the peritoeum, accompanied by large quantities of blood. Here the ovum acts the part of a foreign body, soon determining an acute inflammatory process about it, which possibly may form a cyst-wall, made up almost wholly of plastic lymph, which completely isolates it from the rest of the peritoneal cavity. If the fœtal cyst ruptures, and the contents escape from the amniotic cavity into the midst of the intestinal mass, a renewal of the inflammation occurs, and the cyst just described forms around it. As a rule, the fœtus perishes at or soon after the time of rupture. With the death of the child, it may be converted into a lithopedion; or, through the blood supply of the connective tissue, it may be preserved for years in its soft integrity.

In all forms of ectopic gestation, the connection between the ovum and the abnormal surface upon which it is engrafted is established by a vital adhesion between the chorionic villi and the tissues with which they come in contact.

*The Uterus in Ectopic Gestation.*—From researches made by Clark, Oldham, Virchow, and others, it would seem fair to conclude that the uterus is enlarged, even in the early stages of ectopic gestation. A decidua forms in its cavity, which is usually expelled during the early stages of gestation en masse, with pain and symptoms of abortion, or it may be discharged in shreds and pieces without symptoms.

### EXTRA-UTERINE PREGNANCY: ITS DIAGNOSIS.

Dr. JOSEPH PRICE, of Philadelphia: The literature of extra-uterine pregnancy is scattered through a vast number of periodicals, and consists for the most part of descriptions of cases occurring in the practice of the writers. The most valuable works are those of Mr. Lawson Tait and Dr. John S. Parry. Mr. Tait is undoubtedly correct in his proposition that all extra-uterine pregnancies are primarily tubal, and that all the so-called varieties depend upon the location of the ovum in the tube and the location of the point of rupture of the tube. The subject may be divided into three classes—*first*, extra-uterine pregnancy before the rupture of the tube; *second*, at the time of rupture of the tube; and *third*, after the rupture of the tube. Rupture of the tube is not synonymous with rupture of the fœtal sac, though they generally occur at the same time. The diagnosis of extra-uterine pregnancy before the rupture of the tube is rarely made, and when it is made is of necessity not positive, because the same set of symptoms may arise from a number of pathological conditions. The symptoms they present are as follows: First, partial or complete cessation of menstruation for one or more periods, generally accompanied by other rational symptoms of pregnancy. Second, pain which is peculiar, being generalty severe, paroxysmal, and long-continued; a sickening pelvic pain which is neither cramp-like nor colicky. These pains, probably caused by the distention of the tube, are like to subside for a time, only to return. Third, the appearance of uterine hæmorrhage, which is again peculiar in that it is usually irregular both as to time and quantity; generally lighter in color than the normal discharge and containing shreds of tissue, which are portions of the decidua vera. The general condition of the vagina and cervix may or may not correspond to normal pregnancy. The uterus is generally enlarged and pushed out of place by a tender or exceedingly painful cystic mass, occupying the position of one or the other of the tubes, and freely movable. A differential diagnosis is extremely uncertain. Care must be taken not to hastily exclude pregnancy, because of the apparent return of the catamenia, nor to conclude that miscarriage has taken place on account of the presence of tissue in the discharge. When the fœtus and placenta die before the rupture of the tube, the difficulty of diagnosis is practically insurmountable. These symptoms continue as the pregnancy advances. The tumor, as it increases in size, causes additional symptoms by pressure on the bladder and rectum. Rupture takes place almost always between the eighth and fourteenth week of pregnancy; in a majority of cases before the twelfth week. Now the symptoms vary somewhat, according to point of rupture in the tube, whether into the peritoneal cavity or below it.

EXTRA-UTERINE PREGNANCY: ITS TREATMENT.

Dr. E. E. Montgomery, of Philadelphia, said, that when asked to take part, he chose the subject of the surgical treatment. While his subsequent study had not induced him to depreciate the value of surgery, it had led him to appreciate more highly than he had before done the possibilities of treatment by electricity.

Treatment must depend upon the form with which we are confronted.

Electricity affords an agent which can be relied upon for the destruction of fœtal life. Its efficacy is questioned, but we cannot accept the dicta of men who are ignorant of the manner in which it is used, or of those who condemn it without a trial. An agent which is capable of destroying the life of mice and insects by passing a current through a vessel of water in which they are placed, should be effective in destroying life when brought in close contact with the fœtal envelope through vagina or rectum, as may be most convenient. Its method of action is not electrolytic; the proper method of use is not by puncture. Those doubting its efficacy, question the diagnosis; but it is improbable that all of the cases quoted are cases of mistaken diagnosis. An agent that will dispel conditions affording the subjective and objective symptoms of ectopic gestation is worthy of further consideration.

In conclusion, he recommended the following plans of procedure:

1. In every form of ectopic gestation, prior to the fourth month, the destruction of life by electricity (Faradic current).

2. Between the fourth and sixth months, destruction of life by electricity, and, some weeks later, laparatomy.

3. In rupture, immediate laparatomy, with removal of sac, contents, and the effused blood.

4. In cases that have passed the sixth month, wait until viability is well established, and perform laparatomy, observing every precaution that separation of the placenta does not occur, close the sac above, and drain through the vagina.

5. In case of death of fœtus, it should be removed by laparatomy a few weeks later.

6. Where the fœtus has become macerated and abcess has formed, its sinus should be enlarged and the fœtal residue removed.

Dr. A. Vander Veer, Albany, said: Much of the literature upon ectopic gestation is very vague. The monograph of Sir Spencer Wells on "Abdominal Tumors" is especially valuable for its clear history of cases, and the concise, yet minute, description of the surgical devices employed.

The abdominal surgeon needs to be perfectly familiar with the points of diagnosis, especially in the early stages.

There is a class of cases in which a diagnosis can be made before rupture, and by the tenth week. Another class gives rise to no symptoms other than those of normal pregnancy before the rupture takes place, as in the thirty-five cases of Tait and the eighteen cases found post-mortem by Formad, of Philadelphia. The first class are such patients as have been under the eye of the gynæcologist, perhaps, for years. He will be conversant with the pelvic conditions and relations. The use of electricity as a fœticidal agent is largely an American method of treatment. The strongest objection to its use seems to be that untoward results follow the operation. The operation for the removal of the product of an ectopic gestation before rupture has taken place is to the surgeon an ideal method.

There remains no foreign body to be disposed of, and no serious after-effects are to be feared. Veit records seven successful cases with no failures. When the symptoms show that rupture has taken place, there can be no doubt that an immediate abdominal section is the only proper thing to do.

When ectopic gestation goes safely beyond the fourth month, danger of rupture is small.

The general tendency of operators is, then, to await spurious labor. Shall we then try to remove a living child, or wait until the placental circulation has ceased? The tendency of operators is growing more and more toward primary laparatomy. In view of our improved technique in doing the operation, I believe we are justified in urging primary laparatomy. In primary laparatomy, the placenta ought to be removed either by ligation or exsection.

Dr. A. LAPTHORN SMITH, of Montreal, read a paper on "Some Minute but Important Details in the Management of the Continuous Current in Gynæcology."

The following officers were elected for the ensuing year: President, Dr. Wm. H. Taylor, Cincinnati; Vice-Presidents, Dr. E. E. Montgomery, Philadelphia, and Dr. J. Henry Carstens, Detroit; Secretary, Dr. William Warren Potter, Buffalo; Treasurer, Dr. X. O. Werder, Pittsburgh; Executive Council, Dr. Thomas Opie, Baltimore; Dr. Clinton Cushing, San Francisco; Dr. Melancthon Storrs, Hartford, and Dr. Byron Stanton, Cincinnati.

The next meeting will be held in Cincinnati.

